# Clinical relevance of and risk factors for HSV-related tracheobronchitis or pneumonia: results of an outbreak investigation

**DOI:** 10.1186/cc6175

**Published:** 2007-11-08

**Authors:** Ilka Engelmann, Jens Gottlieb, Astrid Meier, Dorit Sohr, Arjang Ruhparwar, Cornelia Henke-Gendo, Petra Gastmeier, Tobias Welte, Thomas Friedrich Schulz, Frauke Mattner

**Affiliations:** 1 Institute of Virology, Medizinische Hochschule Hannover, Carl-Neuberg-Strasse 1, 30625 Hannover, Germany; 2 Department of Pneumology, Medizinische Hochschule Hannover, Carl-Neuberg-Strasse 1, 30625 Hannover, Germany; 3 Institute of Medical Microbiology and Hospital Epidemiology, Medizinische Hochschule Hannover, Carl-Neuberg-Strasse 1, 30625 Hannover, Germany; 4 Institute of Hygiene and Hospital Epidemiology, Charité, Hindenburgdamm 27 12203 Berlin, Germany; 5 Division of Thoracic and Cardiovascular Surgery, Medizinische Hochschule Hannover, Carl-Neuberg-Strasse 1, 30625 Hannover, Germany; 6 Department of Cardiac Surgery, University of Heidelberg, Im Neuenheimer Feld 110, 69120 Heidelberg, Germany

## Abstract

**Introduction:**

Herpes simplex virus (HSV) type 1 was identified in respiratory specimens from a cluster of eight patients on a surgical intensive care unit within 8 weeks. Six of these patients suffered from HSV-related tracheobronchitis and one from HSV-related pneumonia only. Our outbreak investigation aimed to determine the clinical relevance of and risk factors associated with HSV-related tracheobronchitis or pneumonia in critically ill patients, and to investigate whether the cluster was caused by nosocomial transmission.

**Methods:**

A retrospective cohort study was performed to identify risk factors for the outcomes of HSV-related tracheobronchitis or pneumonia and death using univariable analysis as well as logistic regression analysis. Viruses were typed by molecular analysis of a fragment of the HSV type 1 glycoprotein G.

**Results:**

The cohort of patients covering the outbreak period comprised 53 patients, including six patients with HSV-related tracheobronchitis and one patient with pneumonia only. HSV-related tracheobronchitis or pneumonia was associated with increased mortality (100% in patients with versus 17.8% in patients without HSV-related tracheobronchitis or pneumonia; *P *< 0.0001). The interaction of longer duration of ventilation and tracheotomy was associated with HSV-related tracheobronchitis or pneumonia in multivariable analysis.

Identical HSV type 1 glycoprotein G sequences were found in three patients and in two patients. The group of three identical viral sequences belonged to a widely circulating strain. The two identical viral sequences were recovered from bronchoalveolar lavages of one patient with HSV-related tracheobronchitis and of one patient without clinical symptoms. These viral sequences showed unique polymorphisms, indicating probable nosocomial transmission.

**Conclusion:**

HSV-related tracheobronchitis or pneumonia is associated with increased mortality in critically ill patients. Care should be taken to avoid nosocomial transmission and early diagnosis should be attempted.

## Introduction

Herpes simplex virus (HSV) is a double-stranded DNA virus occurring in two types, HSV-1 and HSV-2. Transmission usually occurs by contact with infected saliva or cutaneous lesions [1]. After primary infection, HSV-1 establishes a life-long latent infection through persistence in neurons of the dorsal root ganglia and the autonomic nervous system [2]. Reactivation can be triggered by local stimuli (ultraviolet irradiation, tissue damage) or by systemic stimuli (fever, menstruation, surgery, physical or emotional stress, hormonal imbalance, immunosuppression) [3]. Clinical manifestations of HSV-1 infection include gingivostomatitis (primary infection), herpes labialis, encephalitis, and keratoconjunctivitis; infections of the respiratory or gastrointestinal tract have been described predominantly in immunosuppressed patients [2].

Asymptomatic shedding of HSV in healthy individuals has been reported to occur in 2–10% of infected individuals [4,5]. HSV-1 can be detected in the upper respiratory tract and in the lower respiratory tract of intensive care unit patients in 22–23% and 16% of cases, respectively [5,6]. Whether these proportions represent clinically relevant HSV infection or, rather, are an indicator of severe disease favouring reactivation without clinical significance is the subject of ongoing debate [5,7-9]. Tracheobronchitis due to HSV has been described in critically ill patients [10,11].

As more than 90% of adults have antibodies specific for HSV-1 [11], infections in adulthood are usually assumed to be reactivation of endogenous virus, although reinfection with a different HSV-1 strain that is immunologically distinct is also possible [12].

Eight patients in a surgical intensive care unit (SICU) had HSV-1 detected in their respiratory tract within 8 weeks. Tracheobronchitis was associated with HSV-1 detection in six patients and with pneumonia in four patients. This cluster prompted us to investigate the clinical impact of HSV-related tracheobronchitis or pneumonia and to identify risk factors predisposing to HSV-related tracheobronchitis or pneumonia and fatal outcome. As the cluster suggested the possibility of nosocomial transmission, molecular epidemiological studies were performed to type all viruses recovered from the patients.

## Materials and methods

### Setting and patients

**Figure 1:**
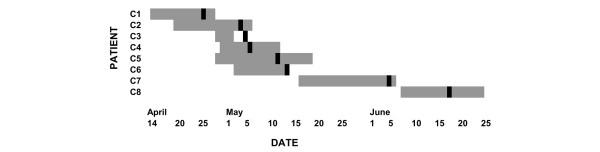
Patient cluster with herpes simplex virus in respiratory specimens on the surgical intensive care unit. Grey bars, period of stay on the surgical intensive care unit; black bars, day when herpes simplex virus type 1 was detected in the respiratory tract of the patient.

When a cluster of six patients with HSV-1-related tracheobronchitis occurred on a 15-bed cardiothoracic SICU (Figure 1), the present outbreak investigation was initiated. Medical records of the SICU and the database of the Department of Virology were reviewed to identify all patients who were hospitalized on this SICU during the time period when the cluster occurred and who had HSV-1 detected (by antigen detection, virus isolation or PCR) in respiratory fluids.

Demographic data as well as underlying diseases, clinical course, any severe clinical presentations in addition to the HSV-1-associated ones and outcome were recorded (Tables 1 and 2). All records of bronchoscopic and radiologic examinations were reassessed, focusing on endobronchial bleedings and lesions or infiltrates compatible with HSV infection. Microbiological and mycological findings were reviewed to determine whether concurrent infections with pathogens other than HSV were present. To assess the clinical relevance of HSV-1 detection in respiratory fluids, the clinical presentations and outcomes of HSV-1-positive patients were analysed (Tables 1 and 2).

**Table 1:** Demographic and clinical characteristics of patients with herpes simplex virus type 1 detection in respiratory specimens

Cluster Patient/Isolate	Gender	Age (years)	Underlying disease	Surgical procedure	Herpes simplex virus-associated infection
C1	Female	81	Aortic stenosis	Replacement of the aortic valve by biological graft	Pneumonia, haemorrhagic tracheobronchitis
C2	Male	74	Infection of the aortic Y-prosthesis	Replacement of the aorta	Haemorrhagic tracheobronchitis
C3	Female	18	Cystic fibrosis	Lung transplantation	-
C4	Male	78	Coronary heart disease	Coronary artery bypass graft	Haemorrhagic tracheobronchitis
C5	Male	61	Coronary heart disease	Coronary artery bypass graft	Pneumonia, haemorrhagic tracheobronchitis
C6	Male	51	Coronary heart disease	Extracorporeal membrane oxygenation implantation	Haemorrhagic tracheobronchitis
C7	Male	67	Covered rupture of an aortic aneurysm	Replacement of the aorta	Pneumonia, haemorrhagic tracheobronchitis
C8	Male	77	Coronary heart disease	Coronary artery bypass graft	Pneumonia

**Table 2:** Clinical and virologic characteristics of patients with herpes simplex virus type 1 detection in respiratory specimens

Cluster Patient/Isolate	Clinical presentation besides herpes simplex virus-associated presentations	Herpes simplex virus detection				Outcome
			
		Specimen type	Direct immunofluorescence testing	Virus culture	PCR	
C1	Right-sided heart failure	Tracheal aspirate	-	-	+	Death
C2	Infection of the aortic Y-prosthesis, intraabdominal bleedings	Bronchoalveolar lavage	+	+	+	Death
C3	-	Bronchoalveolar lavage	+	-	+	Survival
C4	Adult respiratory distress syndrome, internal carotid artery stenosis	Tracheal aspirate	+	+	+	Death
C5	Adult respiratory distress syndrome	Nasopharyngeal swab	+	+	+	Death
C6	-	Bronchoalveolar lavage	+	+	+	Death
C7	Peritonitis with coagulase-negative Staphylococci	Bronchoalveolar lavage	+	+	+	Death
C8	Sepsis	Bronchoalveolar lavage	+	+	+	Death

Bronchoscopies were sampled after routine disinfection. DNA was isolated and used as the input in the diagnostic HSV PCR and in the typing PCR (see below).

The institutional review board approved the outbreak investigation.

### Cohort study

A retrospective cohort study was performed including the 8-week period that entirely covered the cluster episode. During this period all patients admitted to the SICU with a stay longer than 72 hours were included (*n *= 53). The analysed outcomes were death and HSV-related tracheobronchitis or pneumonia. The latter was defined as HSV detection in the respiratory tract concomitant with the presence of tracheobronchitis or pneumonia and an absence of other respiratory pathogens even though histopathology was not performed. Sampling for HSV detection in respiratory fluids was performed if clinically indicated (that is, in case of unexplained deterioration of respiratory function, tracheobronchial bleeding or suspicious mucosal lesions on bronchoscopic examination).

Of the 53 patients, seven fulfilled the criteria for the outcome HSV-related tracheobronchitis or pneumonia. One of the patients with HSV detection in bronchoalveolar lavage was excluded from the analysis of the cohort study (Patient C3) because he did not show symptoms related to HSV (Tables 1 and 2). The HSV glycoprotein G sequence of this patient was included in the molecular epidemiological analysis (see below). None of the remaining 45 patients presented with symptoms of tracheobronchitis. Eight of the 45 remaining patients were confirmed negative for HSV in their lower respiratory tract secretions, whereas the other patients were not tested because testing was only performed if clinical symptoms were evocative.

The variables analysed as risk factors for HSV-related tracheobronchitis or pneumonia and fatal outcome are presented in Tables 3 and 4. The variables pneumonia and HSV-related tracheobronchitis or HSV-related pneumonia were included additionally as risk factors for fatal outcome. As appropriate to analyse the time period at risk for developing HSV-related tracheobronchitis or pneumonia, variables were only regarded as positive if they occurred prior to HSV detection (for patients with HSV-related tracheobronchitis or pneumonia) or prior to SICU discharge (for patients without HSV-related tracheobronchitis or pneumonia).

**Table 3:** Frequency of outcome herpes simplex virus type 1 (HSV-1)-related tracheobronchitis or pneumonia depending on patient characteristics and extrinsic risk factors

Risk factor	Number (%) of patients		Number (%) of patients with HSV-1-related tracheobronchitis or pneumonia		*P *value^a^	Relative risk
			
	Without risk factor	With risk factor	Without risk factor	With risk factor		
Age ≥ median (64 years)	27 (51.9)	25 (48.1)	2 (7.4)	5 (20.0)	0.24	2.70
Gender, male	15 (28.8)	37 (71.2)	1 (6.6)	6 (16.2)	0.66	2.43
Simplified Acute Physiology Score > median (31)	27 (51.9)	25 (48.1)	2 (7.4)	5 (20.0)	0.24	2.70
Time at risk on SICU > median (8.5 days)	26 (50.0)	26 (50.0)	1 (3.9)	6 (23.1)	0.10	6.00
Ventilation time > median (4.7 days)^b^	26 (50.0)	26 (50.0)	0 (0)	7 (26.9)	0.01	-
Bronchoscopy	24 (46.1)	28 (53.9)	3 (12.5)	4 (14.3)	1.00	1.14
Number of bronchoscopies > median (1)	37 (71.1)	15 (28.9)	4 (10.8)	3 (20.0)	0.40	1.85
Tracheotomy	46 (88.5)	6 (11.5)	4 (8.7)	3 (50.0)	0.03	5.75
Reintubation	37 (71.1)	15 (28.9)	4 (10.8)	3 (20.0)	0.40	1.85
Reanimation	44 (84.6)	8 (15.4)	5 (11.4)	2 (25.0)	0.29	2.20
Underlying disease						
Cardiomyopathy^b^	48 (92.3)	4 (7.7)	7 (14.6)	0 (0)	1.00	-
Valvular heart disease	35 (67.3)	17 (32.7)	6 (17.1)	1 (5.9)	0.40	0.34
Cystic fibrosis^b^	51 (98.1)	1 (1.9)	7 (13.7)	0 (0)	1.00	-
Infrarenal aortic aneurysm	47 (90.4)	5 (9.6)	6 (12.8)	1 (20.0)	0.53	1.57
Suprararenal aortic aneurysm	47 (90.4)	5 (9.6)	6 (12.8)	1 (20.0)	0.53	1.57
Coronary heart disease	37 (71.1)	15 (28.9)	3 (8.11)	4 (26.7)	0.17	3.29
Congenital valvular heart disease^b^	48 (92.3)	4 (7.7)	7 (14.6)	0 (0)	1.00	-
Surgical intervention						
Solid organ transplantation^b^	44 (84.6)	8 (15.4)	7 (19.9)	0 (0)	0.58	-
Left ventricular assist device	43 (82.7)	9 (17.3)	5 (11.6)	2 (22.2)	0.59	1.91
Coronary artery bypass graft	38 (73.1)	14 (26.9)	4 (10.5)	3 (21.4)	0.37	2.04
Aortic surgery	41 (78.8)	11 (21.2)	5 (12.2)	2 (18.2)	0.63	1.49
Valve surgery	37 (71.1)	15 (28.9)	6 (16.2)	1 (6.7)	0.66	0.41
Immunosuppressive medication	27 (51.9)	25 (48.1)	5 (18.5)	2 (8.0)	0.42	0.43
Steroids	28 (53.8)	24 (46.2)	5 (17.9)	2 (8.3)	0.43	0.47
Cyclosporin^b^	46 (88.5)	6 (11.5)	7 (15.2)	0 (0)	0.58	-
Mycophenolate mofetil^b^	44 (84.6)	8 (15.4)	7 (15.9)	0 (0)	0.58	-
Basiliximab^b^	46 (88.5)	6 (11.5)	7 (15.2)	0 (0)	0.58	-
Tacrolimus	48 (92.3)	4 (7.7)	6 (12.5)	1 (25.0)	0.45	2.00
Blood products^b^	2 (3.8)	50 (96.2)	0 (0)	7 (14.0)	1.00	-
Erythrocyte concentrates^b^	8 (15.4)	44 (84.6)	0 (0)	7 (15.9)	0.58	-
Fresh frozen plasma^b^	4 (7.7)	48 (92.3)	0 (0)	7 (14.6)	1.00	-
Thrombocyte concentrates	15 (28.8)	37 (71.2)	2 (13.3)	5 (13.5)	1.00	1.01
Number of erythrocyte concentrates > median (7)	27 (51.9)	25 (48.1)	1 (3.7)	6 (24.0)	0.05	6.48
Number of fresh frozen plasma units > median (11.5)	26 (50.0)	26 (50.0)	1 (3.9)	6 (23.1)	0.09	6.00
Number of thrombocyte concentrates > median (3)	26 (50.0)	26 (50.0)	3 (11.5)	4 (15.4)	1.00	1.33
Clotting factor substitution	31 (59.6)	21 (40.4)	2 (6.5)	5 (23.8)	0.10	3.69
C1 esterase inhibitor^b^	49 (94.2)	3 (5.8)	7 (14.3)	0 (0)	1.00	-
Interactions						
Ventilation time > median with tracheotomy	48 (92.3)	4 (7.7)	4 (8.3)	3 (75.0)	0.006	9.00

^a^Fisher's exact test. ^b^Variable could not be included in the logistic regression model for mathematical reasons.

**Table 4:** Frequency of outcome death depending on patient characteristics and extrinsic risk factors

Risk factor	Number (%) of patients		Number (%) of patients with outcome death		*P *value^a^	Relative risk
			
	Without risk factor	With risk factor	Without risk factor	With risk factor		
Age ≥ median (64 years)	27 (51.9)	25 (48.1)	8 (29.6)	7 (28.0)	1.00	0.95
Gender, male	15 (28.8)	37 (71.2)	4 (26.7)	11 (29.7)	1.00	1.12
Simplified Acute Physiology Score > median (31)	27 (51.9)	25 (48.1)	6 (22.2)	9 (36.0)	0.36	1.62
Time at risk on SICU > median (8.5 days)	26 (50.0)	26 (50.0)	5 (19.2)	10 (38.5)	0.22	2.00
Ventilation time > median (4.7 days)	26 (50.0)	26 (50.0)	2 (7.7)	13 (50.0)	0.002	6.50
Bronchoscopy	24 (46.1)	28 (53.9)	3 (12.5)	12 (42.9)	0.03	3.43
Number of bronchoscopies > median (1)	37 (71.1)	15 (28.9)	10 (27.0)	5 (33.3)	0.74	1.23
Tracheotomy	46 (88.5)	6 (11.5)	11 (23.9)	4 (66.7)	0.05	2.79
Reintubation	37 (71.1)	15 (28.9)	10 (27.0)	5 (33.3)	0.74	1.23
Reanimation	44 (84.6)	8 (15.4)	12 (27.3)	3 (37.5)	0.68	1.38
Cardiovascular disease						
Cardiomyopathy^b^	48 (92.3)	4 (7.7)	15 (31.3)	0 (0)	0.31	-
Valvular heart disease	35 (67.3)	17 (32.7)	11 (31.4)	4 (23.5)	0.75	0.75
Cystic fibrosis^b^	51 (98.1)	1 (1.9)	15 (29.4)	0 (0)	1.00	-
Infrarenal aortic aneurysm	47 (90.4)	5 (9.6)	13 (27.7)	2 (40.0)	0.62	1.45
Suprararenal aortic aneurysm	47 (90.4)	5 (9.6)	14 (29.8)	1 (20.0)	1.00	0.67
Coronary heart disease	37 (71.1)	15 (28.9)	8 (21.6)	7 (46.7)	0.10	2.16
Congenital valvular heart disease	48 (92.3)	4 (7.7)	14 (29.2)	1 (25.0)	1.00	0.86
Surgical intervention						
Solid organ transplantation	44 (84.6)	8 (15.4)	14 (31.8)	1 (12.5)	0.41	0.39
Left ventricular assist device	43 (82.7)	9 (17.3)	12 (27.9)	3 (33.3)	0.71	1.19
Coronary artery bypass graft	38 (73.1)	14 (26.9)	9 (23.7)	6 (42.9)	0.19	1.81
Aortic surgery	41 (78.8)	11 (21.2)	12 (29.3)	3 (27.3)	1.00	0.93
Valve surgery	37 (71.1)	15 (28.9)	12 (32.4)	3 (20.0)	0.51	0.62
Immunosuppression	27 (51.9)	25 (48.1)	9 (33.3)	6 (24.0)	0.55	0.72
Steroids	28 (53.8)	24 (46.2)	10 (35.7)	5 (20.8)	0.36	0.58
Cyclosporin	46 (88.5)	6 (11.5)	14 (30.4)	1 (16.7)	0.66	0.55
Mycophenolate mofetil	44 (84.6)	8 (15.4)	14 (31.8)	1 (12.5)	0.41	0.39
Basiliximab	46 (88.5)	6 (11.5)	14 (30.4)	1 (16.7)	0.66	0.55
Tacrolimus	48 (92.3)	4 (7.7)	14 (29.2)	1 (25.0)	1.00	0.86
Blood products^b^	2 (3.8)	50 (96.2)	0 (0)	15 (30.0)	1.00	-
Erythrocyte concentrates	8 (15.4)	44 (84.6)	1 (12.5)	14 (31.8)	0.41	2.55
Fresh frozen plasma^b^	4 (7.7)	48 (92.3)	0 (0)	15 (31.3)	0.31	-
Thrombocyte concentrates	15 (28.8)	37 (71.2)	2 (13.3)	13 (35.1)	0.18	2.64
Number of erythrocyte concentrates > median (7)	27 (51.9)	25 (48.1)	4 (14.8)	11 (44.0)	0.03	2.97
Number of fresh frozen plasma units > median (11.5)	26 (50.0)	26 (50.0)	4 (15.4)	11 (42.3)	0.06	2.75
Number of thrombocyte concentrates > median (3)	26 (50.0)	26 (50.0)	4 (15.4)	11 (42.3)	0.06	2.75
Clotting factors	31 (59.6)	21 (40.4)	6 (19.4)	9 (42.9)	0.12	2.21
C1 esterase inhibitor	49 (94.2)	3 (5.8)	13 (26.5)	2 (66.7)	0.20	2.51
Herpes simplex virus-related tracheobronchitis or pneumonia^b^	45 (86.5)	7 (13.5)	8 (17.8)	7 (100)	<0.0001	-
Pneumonia	46 (88.5)	6 (11.5)	11 (23.9)	4 (66.7)	0.05	2.79

^a^Fisher's exact test. ^b^Variable could not be included in the logistic regression model for mathematical reasons.

### Statistical analysis

Univariable analysis and logistic regression with stepwise (forward and backward) variable selection were performed using SAS software (SAS Institute, Inc., Cary, NC, USA). *P *< 0.05 was regarded as significant for univariable analysis. A significance level of 0.1 was chosen for inclusion of variables into a logistic regression model and for remaining included in the model.

The area under the receiver operating characteristic curve (*c *index) is used to evaluate the predictive power of the logistic regression model. The *c *index represents the probability that the regression model equation assigns randomly chosen patients with HSV higher probabilities of acquiring HSV than randomly chosen patients without HSV [13].

### Viral diagnostics and molecular typing

Respiratory specimens (bronchoalveolar lavage or tracheal aspirates in most cases, nasopharyngeal swab in one patient) were submitted to direct immunofluorescence staining with antibodies specific for HSV. A sample (1–2 ml) of the specimen was added to cell cultures of Hep-2 and Vero cells, and was monitored twice weekly for up to 3 weeks for emergence of a cytopathic effect. DNA was isolated and a real-time PCR was performed, detecting a 254 bp fragment of the HSV UL27 gene (Engelmann, I., Petzold, D.R., Kosinska A., Hepkema B.G., Schulz, T. F. and Heim, A., accepted for publication in Journal of Medical Virology; Title: Rapid quantitative PCR assays for the simultaneous detection of herpes simplex virus, varicella zoster virus, cytomegalovirus, Epstein Barr virus and human herpesvirus 6 DNA in blood and other clinical specimens). Melting curve analysis was used to differentiate HSV-1 and HSV-2.

All viruses recovered from patients on the SICU during the time period of the cluster (designated C; Figure 1) – including patients with HSV-related tracheobronchitis or pneumonia and one patient with asymptomatic HSV detection – were typed. Additionally, HSV isolates from patients of the same SICU outside the cluster period (designated I) and HSV isolates from patients on other wards (designated M) were typed. A hypervariable part of the HSV-1 glycoprotein G was amplified as described by Rekabdar and colleagues [14]. Direct sequencing was performed using the PCR primers and the dRhodamine Terminator Cycle Sequencing Ready Reaction Kit (Applied Biosystems, Foster City, CA, USA) according to the manufacturer's instructions on the ABI PRISM 310 Genetic Analyzer (Applied Biosystems, Foster City, CA, USA). If direct sequencing was unsuccessful the samples were subjected to a nested PCR. The first round involved the amplification of a 600 bp fragment using the outer primers HSV-N1-For (5'-GGGTTCCCACCAACGTCTCC) and HSV-N1-Rev (5'-GGGTGTGTGCGTCGCCCGC). The resulting PCR product was used as template in the PCR described above.

The nucleotide sequences have been submitted to the NCBI database GenBank (accession numbers EF376300–EF376333).

Phylogenetic analysis of the 309 bp sequence of the PCR product was conducted with Phylip software (version 3.63) and with the MEGA Software package (version 3.1) [15]. The phylogenetic tree was constructed using the neighbour-joining method (Kimura two-parameter matrix) with a transition/transversion ratio of 2.0. Bootstrapping was performed with 1,000 replicates and values above 80% are indicated. The tree is presented as an unrooted tree because the use of HSV-2 as an outgroup was impossible due to highly divergent sequences between HSV-1 and HSV-2 in the analysed region.

## Results

HSV-related tracheobronchitis was diagnosed in six patients on a SICU within 8 weeks. Endobronchial bleeding was life-threatening in three patients. HSV-1 was detectable in the respiratory specimens of all patients (Tables 1 and 2) whereas no concurrent bacterial or fungal pathogens were isolated. Furthermore, HSV-1 was detected in bronchoalveolar lavages of two additional patients who did not suffer from HSV-related tracheobronchitis, one of whom had pneumonia (Figure 1). The only surviving patient (C3) had neither clinical nor bronchoscopic signs of HSV-related tracheobronchitis but did receive antiviral medication.

All patients were HSV IgG-positive at the time of HSV detection, suggesting that none of them suffered from primary infection.

### Cohort study

In order to confirm that HSV-related tracheobronchitis or pneumonia was associated with a higher mortality, and to identify risk factors for this entity, a cohort study including 52 patients (after exclusion of one patient with HSV detection in bronchoalveolar lavage but without symptoms related to HSV) was performed. Tables 3 and 4 present the results of univariable analysis for the outcomes HSV-related tracheobronchitis or pneumonia and death. Multiple logistic regression analysis revealed statistical association of HSV-related tracheobronchitis or pneumonia with the interaction between longer duration of ventilation and tracheotomy, and statistical association of HSV-related tracheobronchitis or pneumonia with the transfusion of a higher number of erythrocyte concentrates (Table 5).

**Table 5:** Risk factors for herpes simplex virus-related tracheobronchitis or pneumonia and fatal outcome: results of multiple logistic regression analysis with stepwise variable selection

Variable	Adjusted odds ratio	95% confidence interval
Outcome herpes simplex virus-related tracheobronchitis or pneumonia		
Interaction of ventilation time > median with tracheotomy	32.8	2.72–1000
Number of erythrocyte concentrates > median (7)	8.16	0.96–226.4
*c *index 0.867		
Outcome death		
Ventilation time > median (4.7 days)	30.7	3.3–892.7
Bronchoscopy	19.8	2.2–536.6
Number of thrombocyte concentrates > median (3)	16.3	2.1–388.7
*c *index 0.899		

Significance level for inclusion of variables in the model and for remaining included in the model was 0.1. (For mathematical reasons, herpes simplex virus-related tracheobronchitis or pneumonia could not be included in the model for outcome death.)

Fatal outcome was associated with longer duration of ventilation, with bronchoscopy and with higher number of thrombocyte concentrate transfusions in multiple logistic regression analysis (Table 5). For mathematical reasons HSV-related tracheobronchitis or pneumonia could not be included as a risk factor in the logistic regression model, although it was the most significant risk factor in univariable analysis (Table 4).

No other examined variable showed a significant association with fatal outcome or with HSV-related tracheobronchitis or pneumonia in multiple logistic regression analysis.

### Molecular epidemiology

**Figure 2:**
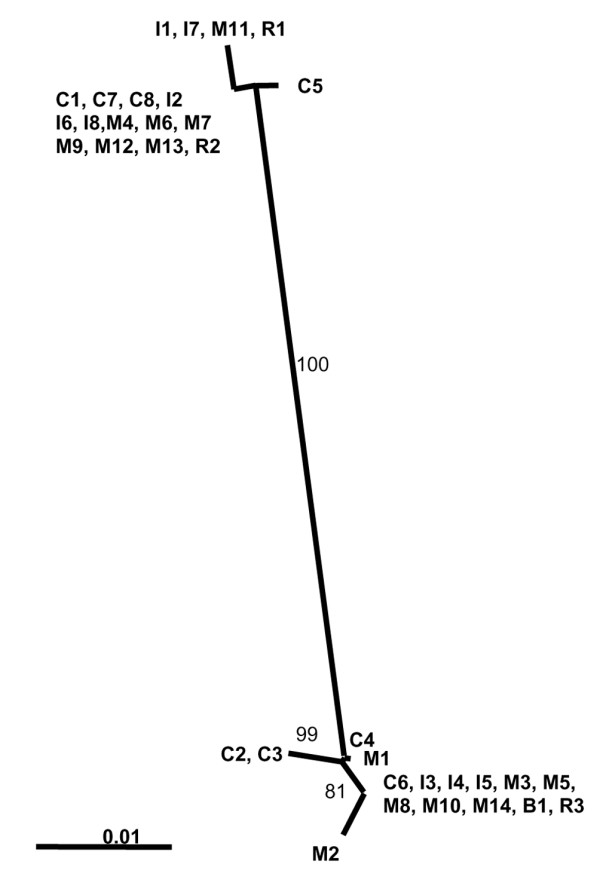
Neighbour-joining tree of partial herpes simplex virus type 1 glycoprotein G sequences. Numbers indicate percentage bootstrap values (only shown if >80). Scale bar indicates 1% genetic divergence. C, cluster patients' viral sequences; I, surgical intensive care unit patients' viral sequences; M, viral sequences from patients from other wards; B, viral sequence recovered from brochoscope; R, reference strains (R1 = 17+, R2 = F, R3 = Kos).

**Figure 3:**
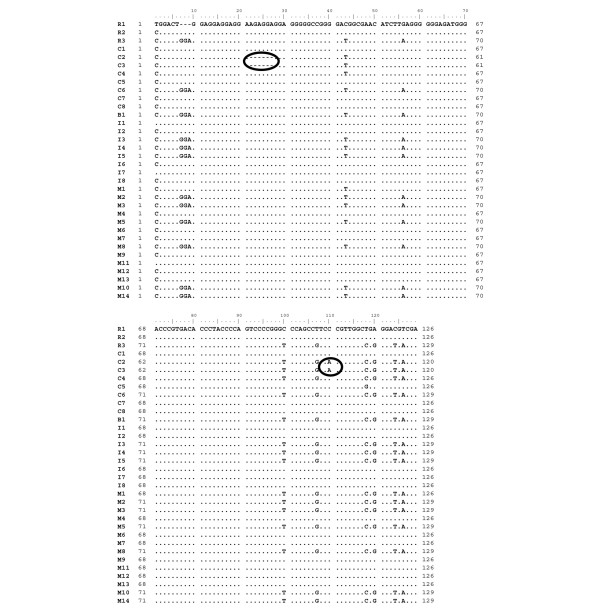
Partial alignment of herpes simplex virus type 1 glycoprotein G sequences. Black circles, unique polymorphisms in viral sequences C2 and C3. C, cluster patients' viral sequences; I, SICU patients' viral sequences; M, viral sequences from patients from other wards; B, viral sequence recovered from brochoscope ; R, reference strains (R1 = 17+, R2 = F, R3 = Kos).

To address the question of endogenous reactivation versus nosocomial transmission, all cluster patients' viruses were subjected to molecular typing. Sequencing of the hypervariable region of the viral glycoprotein G revealed that patients' viruses fell into two groups, with viral sequences of patients C2 and C3 grouping together and with viral sequences of patients C1, C7 and C8 grouping together (Figures 2 and 3). Three cluster patients' viral isolates showed HSV-1 sequences that were not detected in other cluster patients' viral isolates (isolates C4, C5 and C6; Figure 2).

As some viral genotypes are known to predominate even in epidemiologically unrelated samples [16], analysis was extended to other HSV isolates obtained during approximately the same time period in the same hospital. Epidemiologically unrelated virus isolates (isolates I2, I6, I8, M4, M6, M7, M9, M12 and M13) that showed sequences identical to those of the cluster patients were found in the group represented by viral sequences C1, C7 and C8 (Figures 2 and 3). Viral sequences of patients C2 and C3, however, showed a unique sequence that was not identified in any of the epidemiologically unrelated isolates and could not be found in the NCBI database. The C2/C3 viral sequence was characterized by a deletion of six base pairs in a repeat region and a point mutation at position 110 (of the alignment of partial glycoprotein G sequences) (Figure 3). Isolate C2 was obtained from a patient with HSV-related tracheobronchitis. In contrast to that, the patient from whom the viral sequence C3 was recovered did not show symptoms associated with HSV detection. Those two patients had overlapping stays on the SICU, and HSV was detected in their bronchoalveolar lavages on consecutive days (Figure 1).

Two out of 11 bronchoscope samples tested positive in the HSV real-time PCR and were also subjected to the HSV glycoprotein G PCR and the nested glycoprotein G PCR. A PCR product and sequence was obtained in only one case (viral sequence B1; Figures 2 and 3). The viral sequence B1 clustered with patient isolate C6 and a group of other isolates not epidemiologically related.

## Discussion

HSV-1 detection in respiratory fluids of critically ill patients is frequently reported but its clinical relevance is often uncertain [5,7-9]. In our report the clinical relevance of HSV-1 detection in the respiratory tract is clearly shown by the fact that six out of eight patients with HSV-1 detection in their respiratory tract presented with haemorrhagic tracheobronchitis and four of the eight patients showed radiologic evidence of pneumonia. Only one out of eight patients had no symptoms or signs consistent with HSV-1 infection (patient C3); this patient received ganciclovir prophylaxis as part of a postlung-transplant care regimen, which might have prevented development of a clinically relevant HSV-1 infection and might be the reason why virus culture was negative. Furthermore, the clinical relevance of HSV-related tracheobronchitis or pneumonia in critically ill patients is shown by its association with significantly increased mortality compared with patients without HSV-related tracheobronchitis or pneumonia (100% versus 17.8%, *P *< 0.0001; Table 4). We therefore conclude that HSV-1 reactivation in the respiratory tract accompanied by tracheobronchitis or pneumonia is clinically relevant and is associated with high mortality. It is not possible, however, to prove to which extent HSV-related tracheobronchitis or pneumonia contributed to the fatal outcome in our patients because all patients suffered from other severe medical conditions.

In agreement with our results a recent study showed that lower respiratory tract infection with HSV was associated with poorer outcome (prolonged duration of mechanical ventilation and intensive care unit stay as well as more episodes of bacterial ventilator-associated pneumonia), although it was not associated with increased mortality in their patient cohort [17]. Interestingly, the clinical picture was different, as tracheobronchial bleeding was not described as one of the predominant symptoms [17].

Antiviral therapy with acyclovir initiated after diagnosis of HSV-1 had been established in five of eight patients on average 2.9 days before death (data not shown). The therapy initiation, however, seems to have been too late to improve outcome. In case of endobronchial bleedings or bronchoscopic signs of haemorrhagic tracheobronchitis, therefore, acyclovir treatment should be initiated directly after bronchoscopy and specimen sampling (prior to diagnosis of HSV-1 infection), which would have been on average 8.6 days prior to death in our collective (data not shown).

Surgical procedures and critical illness result in immune dysfunction [18,19], which can be regarded as a predisposing factor for HSV reactivation in the patients described here. The vagal ganglia are thought to be the source of lower respiratory and oesophageal HSV reactivation [20]. Mucosal damage caused by intubation and mechanical ventilation, thoracic surgery or aspiration has been hypothesized to favour HSV reactivation [5,11,17,21,22]. Our observation that the interaction between longer duration of ventilation and tracheotomy (which was performed under bronchoscopic assistance) was a risk factor for HSV-related tracheobronchitis or pneumonia in logistic regression analysis may be explained by inoculation of HSV-1-positive fluids from the upper respiratory tract via bronchoscopically assisted tracheotomy or by mechanical airway irritation as a possible stimulus of reactivation. Whether longer duration of ventilation or tracheotomy alone represent significant risk factors for HSV-related tracheobronchitis or pneumonia remains unclear because they were not identified as independent risk factors in logistic regression analysis, and we cannot exclude that the interaction between longer duration of ventilation and tracheotomy indicates the severity of disease.

The most significant risk factor for fatal outcome identified in univariable analysis was HSV-related tracheobronchitis or pneumonia (*P *< 0.0001, Table 4). Owing to the fact that all patients with HSV-related tracheobronchitis or pneumonia died, this factor could not be included in the logistic regression model for mathematical reasons. Longer duration of ventilation, bronchoscopy and higher number of thrombocyte concentrate transfusions were associated with fatal outcome in the logistic regression analysis. These variables might be surrogate markers for more severe illness rather than risk factors.

Our investigation has the following limitations. Owing to initiation of the analysis in a suspected outbreak situation, systematic testing of respiratory samples for HSV was not performed for all patients. The outcome was therefore defined clinically (that is, presence or absence of HSV-related tracheobronchitis or pneumonia) and not virologically (absence of HSV detection). HSV pneumonia or HSV-related tracheobronchitis was not confirmed by histopathology because, clinically, the presence of pneumonia or tracheobronchitis concurrent with HSV detection in respiratory fluids in the absence of other respiratory pathogens was regarded as sufficient for diagnosis and initiation of antiviral treatment. The size of the cohort is rather small, a limitation that is inherent to outbreak investigations.

Worthy of note is the fact that two bronchoscope samples had HSV-1 DNA detected after routine disinfection procedures. Typing was successful in only one case (viral sequence B1), which had a sequence identical to patient isolate C6 and to a number of epidemiologically unrelated virus isolates (Figures 2 and 3). The bronchoscope sample B1, however, was collected more than 2 months later than the positive specimen of patient isolate C6. An epidemiological association is therefore unlikely. Furthermore, as no viable virus was recovered in virus culture, the significance of this finding concerning the possibility of transmission is uncertain. Testing of bronchoscope samples was initiated when the cluster was already recognized and first preventive measures had already been implemented. We therefore could not clarify whether bronchoscopes were the source of the suspected nosocomial transmission. Bronchoscopes, however, have been identified as sources of nosocomial transmission of other pathogens [23-26].

Studies examining HSV-1 detection in the respiratory tract of intensive care unit patients have assumed HSV-1 positivity to be caused by endogenous reactivation, and have not considered transmission although viruses were not typed [5,7-9]. HSV infections caused by nosocomial transmission, however, have been reported in different settings [27-35]. In those studies, viruses were typed by restriction fragment length polymorphism and almost identical restriction patterns have been interpreted as proof of transmission. That approach, however, does not take into account that some viral genotypes predominate in epidemiologically unrelated virus isolates [16]. Taking this fact into consideration we extended our sequence-based typing to epidemiologically unrelated virus isolates from the same hospital.

We found three cluster patients with HSV-1 sequences that were not detected in other cluster patients' viral isolates (isolates C4, C5 and C6; Figure 2). This finding favours endogenous reactivation as an underlying mechanism.

The group of three cluster patients with identical viral sequences also showed identical sequences to nine epidemiologically unrelated isolates and to the reference strain F (Figure 2). Furthermore, the epidemiological association between these three patients is weak (patient C1 stayed on the SICU at the beginning of the cluster, and patients C7 and C8 at the end; Figure 1). These findings favour the hypothesis that their viruses are part of the predominating genotypes. One patient pair (patients C2 and C3) that had a strong epidemiological association (overlapping stay in the SICU, HSV diagnosis on consecutive days; Figure 1) showed unique polymorphisms in the HSV glycoprotein G sequence that were not identified in any other sequenced isolates nor in the NCBI database. In this case we assume transmission to be likely. As both patients were IgG-positive prior to HSV detection this could represent nosocomial reinfection with a different virus strain. Patient C3 did not present with HSV-related tracheobronchitis but rather with asymptomatic HSV infection, possibly because the ganciclovir prophylaxis he received prevented development of a clinically relevant HSV infection.

As HSV isolates are not routinely typed it is possible that nosocomial transmission is not a rare event but might often not be recognized as such.

## Conclusion

In the present article we have shown that HSV-related tracheobronchitis or pneumonia represents an important infectious complication in critically ill patients. This differential diagnosis has to be considered especially in the case of tracheobronchial haemorrhage or if the patient's condition does not improve with antibacterial and antifungal therapy. Whether acyclovir prophylaxis or early treatment in cases suspicious for HSV-related tracheobronchitis or pneumonia in critically ill patients has the potential of preventing fatal outcome should be addressed in future prospective studies.

## Key messages

• HSV-related tracheobronchitis or pneumonia is associated with high mortality in critically ill patients.

• Tracheobronchial haemorrhage should prompt diagnostics for HSV.

• Molecular typing of virus isolates revealed one event of probable nosocomial transmission.

## Abbreviations

bp = base pair; HSV = herpes simplex virus; SICU = surgical intensive care unit; PCR = polymerase chain reaction.

## Competing interests

The authors declare that they have no competing interests.

## Authors' contributions

IE participated in the design of the outbreak investigation, carried out the molecular genetic studies and the phylogenetic analysis, and wrote the manuscript. JG participated in the collection, interpretation and analysis of clinical data. AM participated in the collection, interpretation and analysis of clinical data. DS performed the statistical analysis. AR participated in the collection, interpretation and analysis of clinical data. CH-G participated in the phylogenetic analysis. PG participated in the study design, interpretation of data and drafting of the manuscript. TW participated in the interpretation of data and drafting of the manuscript. TFS participated in the study design, interpretation of the data and drafting of the manuscript. FM initiated the study, participated in the design of the study and in the interpretation and analysis of clinical data, and contributed to the writing of the manuscript.

## References

[B1] Adler SP, Marshall G (2004). Herpes simplex virus. Hospital Epidemiology and Infection Control.

[B2] Whitley RJ, Roizman B (2001). Herpes simplex virus infections. Lancet.

[B3] Whitley RJ, Knipe DM, Howley PM (2001). Herpes simplex viruses. Virology.

[B4] Hatherley LI, Hayes K, Jack I (1980). Herpes virus in an obstetric hospital. II: asymptomatic virus excretion in staff members. Med J Aust.

[B5] Bruynseels P, Jorens PG, Demey HE, Goossens H, Pattyn SR, Elseviers MM, Weyler J, Bossaert LL, Mentens Y, Ieven M (2003). Herpes simplex virus in the respiratory tract of critical care patients: a prospective study. Lancet.

[B6] Cook CH, Martin LC, Yenchar JK, Lahm MC, McGuinness B, Davies EA, Ferguson RM (2003). Occult herpes family viral infections are endemic in critically ill surgical patients. Crit Care Med.

[B7] Cook CH, Yenchar JK, Kraner TO, Davies EA, Ferguson RM (1998). Occult herpes family viruses may increase mortality in critically ill surgical patients. Am J Surg.

[B8] Ong GM, Lowry K, Mahajan S, Wyatt DE, Simpson C, O'Neill HJ, McCaughey C, Coyle PV (2004). Herpes simplex type 1 shedding is associated with reduced hospital survival in patients receiving assisted ventilation in a tertiary referral intensive care unit. J Med Virol.

[B9] van den Brink JW, Simoons-Smit AM, Beishuizen A, Girbes AR, Strack van Schijndel RJ, Groeneveld AB (2004). Respiratory herpes simplex virus type 1 infection/colonisation in the critically ill: marker or mediator?. J Clin Virol.

[B10] Sherry MK, Klainer AS, Wolff M, Gerhard H (1988). Herpetic tracheobronchitis. Ann Intern Med.

[B11] Klainer AS, Oud L, Randazzo J, Freiheiter J, Bisaccia E, Gerhard H (1994). Herpes simplex virus involvement of the lower respiratory tract following surgery. Chest.

[B12] Roest RW, Carman WF, Maertzdorf J, Scoular A, Harvey J, Kant M, van der Meijden WI, Verjans GM, Osterhaus AD (2004). Genotypic analysis of sequential genital herpes simplex virus type 1 (HSV-1) isolates of patients with recurrent HSV-1 associated genital herpes. J Med Virol.

[B13] Hanley JA, McNeil BJ (1982). The meaning and use of the area under a receiver operating characteristic (ROC) curve. Radiology.

[B14] Rekabdar E, Tunback P, Liljeqvist JA, Bergstrom T (1999). Variability of the glycoprotein G gene in clinical isolates of herpes simplex virus type 1. Clin Diagn Lab Immunol.

[B15] Kumar S, Tamura K, Nei M (2004). MEGA3: integrated software for molecular evolutionary genetics analysis and sequence alignment. Brief Bioinform.

[B16] Sakaoka H, Kurita K, Iida Y, Takada S, Umene K, Kim YT, Ren CS, Nahmias AJ (1994). Quantitative analysis of genomic polymorphism of herpes simplex virus type 1 strains from six countries: studies of molecular evolution and molecular epidemiology of the virus. J Gen Virol.

[B17] Luyt CE, Combes A, Deback C, Aubriot-Lorton MH, Nieszkowska A, Trouillet JL, Capron F, Agut H, Gibert C, Chastre J (2007). Herpes simplex virus lung infection in patients undergoing prolonged mechanical ventilation. Am J Respir Crit Care Med.

[B18] Howard RJ, Simmons RL (1974). Acquired immunologic deficiencies after trauma and surgical procedures. Surg Gynecol Obstet.

[B19] Slade MS, Simmons RL, Yunis E, Greenberg LJ (1975). Immunodepression after major surgery in normal patients. Surgery.

[B20] Tuxen DV (1994). Prevention of lower respiratory herpes simplex virus infection with acyclovir in patients with adult respiratory distress syndrome. Chest.

[B21] Hanley PJ, Conaway MM, Halstead DC, Rhodes LV, Reed J (1993). Nosocomial herpes simplex virus infection associated with oral endotracheal intubation. Am J Infect Control.

[B22] Camps K, Jorens PG, Demey HE, Pattyn SR, Ieven M (2002). Clinical significance of herpes simplex virus in the lower respiratory tract of critically ill patients. Eur J Clin Microbiol Infect Dis.

[B23] Corne P, Godreuil S, Jean-Pierre H, Jonquet O, Campos J, Jumas-Bilak E, Parer S, Marchandin H (2005). Unusual implication of biopsy forceps in outbreaks of *Pseudomonas aeruginosa *infections and pseudo-infections related to bronchoscopy. J Hosp Infect.

[B24] Bou R, Aguilar A, Perpinan J, Ramos P, Peris M, Lorente L, Zuniga A (2006). Nosocomial outbreak of *Pseudomonas aeruginosa *infections related to a flexible bronchoscope. J Hosp Infect.

[B25] Sorin M, Segal-Maurer S, Mariano N, Urban C, Combest A, Rahal JJ (2001). Nosocomial transmission of imipenem-resistant *Pseudomonas aeruginosa *following bronchoscopy associated with improper connection to the Steris System 1 processor. Infect Control Hosp Epidemiol.

[B26] Michele TM, Cronin WA, Graham NM, Dwyer DM, Pope DS, Harrington S, Chaisson RE, Bishai WR (1997). Transmission of *Mycobacterium tuberculosis *by a fiberoptic bronchoscope. Identification by DNA fingerprinting. JAMA.

[B27] Francis DP, Herrmann KL, MacMahon JR, Chavigny KH, Sanderlin KC (1975). Nosocomial and maternally acquired herpesvirus hominis infections. A report of four fatal cases in neonates. Am J Dis Child.

[B28] Linnemann CC, Buchman TG, Light IJ, Ballard JL (1978). Transmission of herpes-simplex virus type 1 in a nursery for the newborn. Identification of viral isolates by D.N.A. 'fingerprinting'. Lancet.

[B29] Adams G, Stover BH, Keenlyside RA, Hooton TM, Buchman TG, Roizman B, Stewart JA (1981). Nosocomial herpetic infections in a pediatric intensive care unit. Am J Epidemiol.

[B30] Hammerberg O, Watts J, Chernesky M, Luchsinger I, Rawls W (1983). An outbreak of herpes simplex virus type 1 in an intensive care nursery. Pediatr Infect Dis.

[B31] Manzella JP, McConville JH, Valenti W, Menegus MA, Swierkosz EM, Arens M (1984). An outbreak of herpes simplex virus type I gingivostomatitis in a dental hygiene practice. JAMA.

[B32] Sakaoka H, Aomori T, Ozaki I, Ishida S, Fujinaga K (1984). Restriction endonuclease cleavage analysis of herpes simplex virus isolates obtained from three pairs of siblings. Infect Immun.

[B33] Van Dyke RB, Spector SA (1984). Transmission of herpes simplex virus type 1 to a newborn infant during endotracheal suctioning for meconium aspiration. Pediatr Infect Dis.

[B34] Sakaoka H, Saheki Y, Uzuki K, Nakakita T, Saito H, Sekine K, Fujinaga K (1986). Two outbreaks of herpes simplex virus type 1 nosocomial infection among newborns. J Clin Microbiol.

[B35] Perl TM, Haugen TH, Pfaller MA, Hollis R, Lakeman AD, Whitley RJ, Nicholson D, Hunter GA, Wenzel RP (1992). Transmission of herpes simplex virus type 1 infection in an intensive care unit. Ann Intern Med.

